# Supplementary material for: Insights into the Cellular Localization and Functional Properties of TSPYL5 Protein
Source: Int J Mol Sci. 2023 Dec 19;25(1):39. doi: 10.3390/ijms25010039 (PMC10779080; doi:10.3390/ijms25010039)

UNIVERSITY OF DUNDEE

NoD
Nucleolar localization sequence Detector

[Home](#)
[Help](#)
[Downloads](#)
[The Barton Group](#)

NoLS predictions for protein sp|Q86VY4|TSYL5_HUMAN
(these predictions are based only on sequence)

One NoLS is predicted in this protein:

MSGRSRGRKSSRAKNRGKGRAKARVRPAPD (between positions 1 and 30)

Position in full-length protein (NoLSs shown in red):

```
MSGRSRGRKSSRAKNRGKGRAKARVRPAPDDAPRDPDPSQYQSLGEDTQAAQVQAGAGWG
GLEAAASAQLLRLGEEAACRLPLDCGLALRARAAGDHGQAAARPGPGKAASLSERLAADT
VFVGTAGTVGRPKNAPRVGNRRGPAGKKAPETCSTAGRGPQVIAGGRQKKGAAGENTSVS
AGEEKKEERDAGSGPPATEGSMDTLENVQLKLENMNAQADRAYLRLSRKFGQLRLQHLER
RNHLIQNIPGFWGQAFQNHPQLASFLNSQEKEVLSYLNSLEVEELGLARLGYKIKFYFDR
NPYFQNKVLIKEYGCGPSGQVVSRSTPIQWLPGHDLQSLSQGNPENNRSFFGWFSNHSSI
ESDKIVEIINEELWPNPLQFYLLSEGARVEKGKEKEGRQGPGKQPMETTQPGVSQSN
```

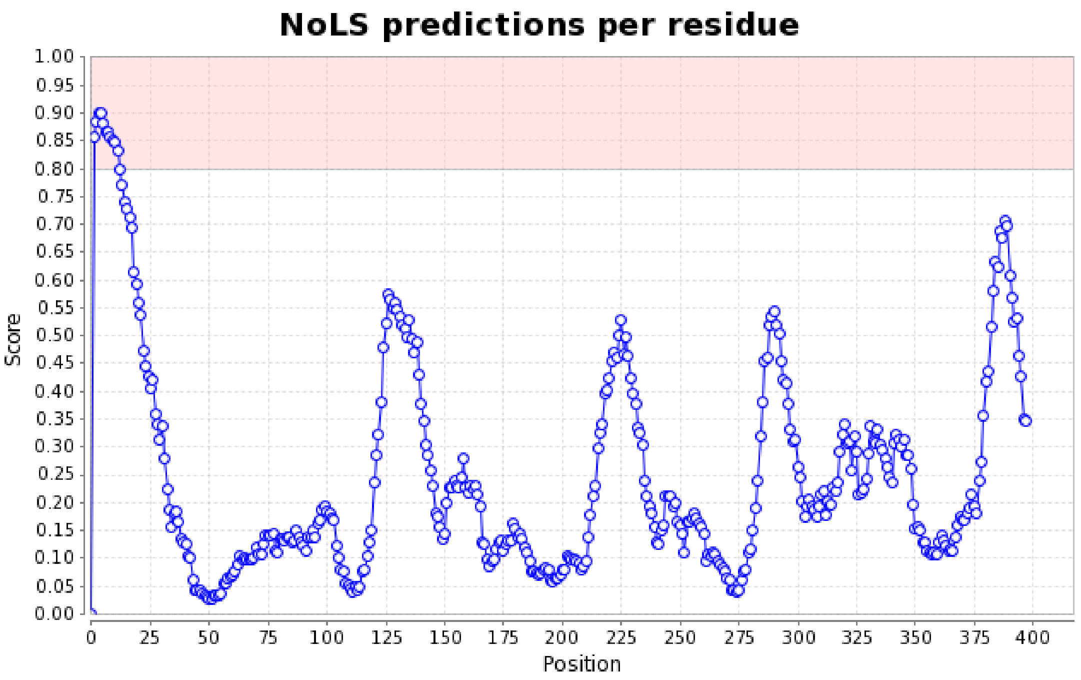

Supplement: Supplementary file 1 [file ijms-25-00039-s001.zip › Figure S1 - NOD prediction.pdf]
