# Supplementary material for: Insights into the Cellular Localization and Functional Properties of TSPYL5 Protein
Source: Int J Mol Sci. 2023 Dec 19;25(1):39. doi: 10.3390/ijms25010039 (PMC10779080; doi:10.3390/ijms25010039)
Supplement: Supplementary file 1 [file ijms-25-00039-s001.zip › Figure S2 - NES prediction.pdf]

NES prediction

1) **NES prediction using LocNES tool**
(http://prodata.swmed.edu/LRNes/predictNES/web_data/LocNES1974202827.php)

chooklab **LocNES**

UT SOUTHWESTERN MEDICAL CENTER

Locating Nuclear Export Signals or NESs

Home | Documentation | NESdb | Chook Lab

Thank you for using LocNES!
Your prediction results are:

Protein Name	Position	Sequence	Score
>LocNES1974202827_0	169-183	QLLRLGEEAACRLPL	0.243
>LocNES1974202827_0	209-223	AASLSERLAADTVFV	0.032
>LocNES1974202827_0	296-310	PATEGSMDTLENVQL	0.015
>LocNES1974202827_0	301-315	SMDTLENVQLKLENM	0.179
>LocNES1974202827_0	321-335	RAYLRLSRKFGQLRL	0.068
>LocNES1974202827_0	368-382	SQEKEVLSYLNSLEV	0.237
>LocNES1974202827_0	373-387	VLSYLNSLEVEELGL	0.201
>LocNES1974202827_0	382-396	VEELGLARLGYKIKF	0.013

Please see Documentation for detailed explanation of your results.

©2014 Yuh Min Chook Laboratory | Department of Pharmacology | UT Southwestern Medical Center

2) **NES prediction using NLSdb**

NLSdb sequence search results

Show 10 entries

Copy CSV

Previous 1 Next

Query	Signal	Signal Type	Start	End	ConfNuc	ConfFam	Annotation Type	Origin	Added	Modified
Q86VY4	VEELGL	NES	282	287	2	2	Potential	In Silico Mutagenesis	2017-10-11	2017-10-11
All	All	All					All	All		

Showing 1 to 1 of 1 entries

Previous 1 Next

* TSPYL5 Uniprot ID - Q86VY4
